# High throughput deep degradome sequencing reveals microRNAs and their targets in response to drought stress in mulberry (*Morus alba*)

**DOI:** 10.1371/journal.pone.0172883

**Published:** 2017-02-24

**Authors:** Ruixue Li, Dandan Chen, Taichu Wang, Yizhen Wan, Rongfang Li, Rongjun Fang, Yuting Wang, Fei Hu, Hong Zhou, Long Li, Weiguo Zhao

**Affiliations:** 1 School of Biology and Technology, Jiangsu University of Science and Technology, Zhenjiang, Jiangsu, P. R. China; 2 The Sericultural Research Institute, Anhui Academy of Agricultural Sciences, Hefei, Anhui, P. R. China; 3 The Plant Protection and Agro-products Safety Institute, Anhui Academy of Agricultural Sciences, Hefei, Anhui, P. R. China; Dokuz Eylul Universitesi, TURKEY

## Abstract

MicroRNAs (miRNAs) play important regulatory roles by targeting mRNAs for cleavage or translational repression. Identification of miRNA targets is essential to better understanding the roles of miRNAs. miRNA targets have not been well characterized in mulberry (*Morus alba*). To anatomize miRNA guided gene regulation under drought stress, transcriptome-wide high throughput degradome sequencing was used in this study to directly detect drought stress responsive miRNA targets in mulberry. A drought library (DL) and a contrast library (CL) were constructed to capture the cleaved mRNAs for sequencing. In CL, 409 target genes of 30 conserved miRNA families and 990 target genes of 199 novel miRNAs were identified. In DL, 373 target genes of 30 conserved miRNA families and 950 target genes of 195 novel miRNAs were identified. Of the conserved miRNA families in DL, mno-miR156, mno-miR172, and mno-miR396 had the highest number of targets with 54, 52 and 41 transcripts, respectively, indicating that these three miRNA families and their target genes might play important functions in response to drought stress in mulberry. Additionally, we found that many of the target genes were transcription factors. By analyzing the miRNA-target molecular network, we found that the DL independent networks consisted of 838 miRNA-mRNA pairs (63.34%). The expression patterns of 11 target genes and 12 correspondent miRNAs were detected using qRT-PCR. Six miRNA targets were further verified by RNA ligase-mediated 5’ rapid amplification of cDNA ends (RLM-5’ RACE). Gene Ontology (GO) annotations and Kyoto Encyclopedia of Genes and Genomes (KEGG) pathway analysis revealed that these target transcripts were implicated in a broad range of biological processes and various metabolic pathways. This is the first study to comprehensively characterize target genes and their associated miRNAs in response to drought stress by degradome sequencing in mulberry. This study provides a framework for understanding the molecular mechanisms of drought resistance in mulberry.

## Introduction

MicroRNAs (miRNAs) are endogenous non-coding small RNAs containing 21–24 nucleotides. miRNAs regulate gene expression accurately and effectively at the post-transcriptional level by repressing translation or directly degrading target mRNAs [1,2]. In plant cells, primary miRNA (pri-miRNA) with a cap and a poly (A) tail is processed into precursors of miRNA (pre-miRNA) containing the distinctive stem-loop structure [3], and thereafter the pre-miRNA is cleaved into a miRNA/miRNA* duplex [4]. Then the miRNA/miRNA* duplex is methylated by Hua Enhancer 1 (HEN1) and transported into the cytoplasm [5]. Mature plant miRNAs are loaded into the ARGONAUTE 1 (AGO1) complex to suppress translation or to cleave the target transcripts [6]. Plant miRNAs have been largely implicated in degradation of their RNA targets by slicing precisely between the 10th and 11th nucleotides (nt) from the 5’ end of miRNAs. Recently, increasing evidence has indicated that miRNAs participate in various processes, such as plant growth, physiological and biochemical processes, signal transduction, cell apoptosis, and biotic and abiotic stress responses [7–11].

To date, hundreds of small RNAs have been isolated by direct cloning and by deep sequencing in higher plants, such as *Arabidopsis thaliana*, rice, soybean, chrysanthemum, and so on [12–14]. As negative regulators of gene expression, plant miRNAs can respond to biotic and abiotic stress [15–17]. By establishing small RNA libraries of *A*. *thaliana* under high salt, drought, ABA and cold treatment, 30 miRNAs associated with abiotic stress were identified [18]. More than 40 plant miRNA family genes are now associated with high salt and drought stress [19,20]. Upon drought treatment, expression of miR159, miR160, miR166, miR169, miR172, miR395, miR396, miR408, miR472, miR477, miR482, miR1858, miR2118, and miR5049 were found to be significantly differentiated in bread wheat [21]. Xie et al found that a series of miRNAs are associated with these top-ranked genes that combat drought and salinity stress in cotton, including miR164, miR172, miR396, miR1520, miR6158, -n24, -n56, and -n59 [22]. Salinity and drought stress induces elevated expression of miR319 in creeping bentgrass, resulting in downregulation of at least four putative target genes of miR319 (AsPCF5, AsPCF6, AsPCF8, and AsTCP14) as well as a homolog of the rice NAC domain gene AsNAC60, and therefore contributes to plant abiotic stress response [23]. Eldem et al found that the expression level of 262 (104 up-regulated, 158 down-regulated) of the 453 miRNAs changed significantly in leaf tissue, whereas 368 (221 up-regulated, 147 down-regulated) of the 465 miRNAs had expression levels that changed significantly in root tissue upon drought stress. The expression level of miR159, miR169, miR393, miR397, miR398 and miR395 had different changes between root and leaf in response to drought [24].

However, few studies have identified miRNAs involved in response to abiotic stress in mulberry [3]. Due to the high degree homology of sequence and function of miRNAs between the different species [25], miRNAs are likely to represent a more primitive physiological regulatory mechanism [26]. Illuminating the function of these tiny molecule nucleotides requires efficient approaches to identify their target genes. Originally, plant miRNA targets were studied via computational prediction, which is based on the perfect sequence complementarity between a miRNA and the target mRNA or sequence conservation among different species [27]. However, targets prediction often has a high level of mismatch in miRNA-mRNA pairs and every single predicted gene must be verified independently. This one-at-a-time isolation of target cleavage remnants is laborious, time-consuming and costly [28,29]. To overcome this limitation, a new method called degradome sequencing has been successfully established to identify small RNA target transcripts at a global scale [30–32]. The method includes deep sequencing, bioinformatic analysis and 5’-rapid amplification of cDNA ends (5’ RACE). It has been used for global identification of miRNA-target RNA pairs in numerous plant species, such as *A*. *thaliana*, *Oryza sativa*, *Glycine max* and *Zea mays* [29,30,33,34].

Drought is one of the major natural disasters affecting plants, and dry weather is becoming more frequent with the intensification of the greenhouse effect. This results in huge losses in industrial and agricultural production, and serious damage to the ecological environment.

Mulberry (*Morus alba*) is an important perennial economic tree, and has a broad ecological distribution in China. It is used in sericulture and has important economic and ecological value. It is highly adaptable to poor environments and can endure salt, drought, cold, waterlogging, and heavy metal ions [35,36]. Certain miRNAs (such as miR159, miR169 and miR398) are known to play an important role in response to drought stress in *A*. *thaliana* [12,16,30]. However, these miRNAs and their molecular roles have not been verified in mulberry. This study was conducted to: (1) comprehensively investigate miRNA targets in response to drought stress in mulberry; (2) analyze the regulatory relationship between miRNAs and their target genes in mulberry through the analysis of miRNA-target molecular network of DL and CL; (3) further verify six of the miRNA targets by RNA ligase-mediated 5’ rapid amplification of cDNA ends (RLM-5’ RACE); and (4) characterize differential expression patterns of 12 drought-responsive miRNAs and target mRNAs in mulberry by stem-loop qRT-PCR. The findings open a new pathway to greatly understand the molecular basis in response to drought stress in mulberry.

## Materials and methods

### Plant materials and drought stress treatment

**Fig 1:**
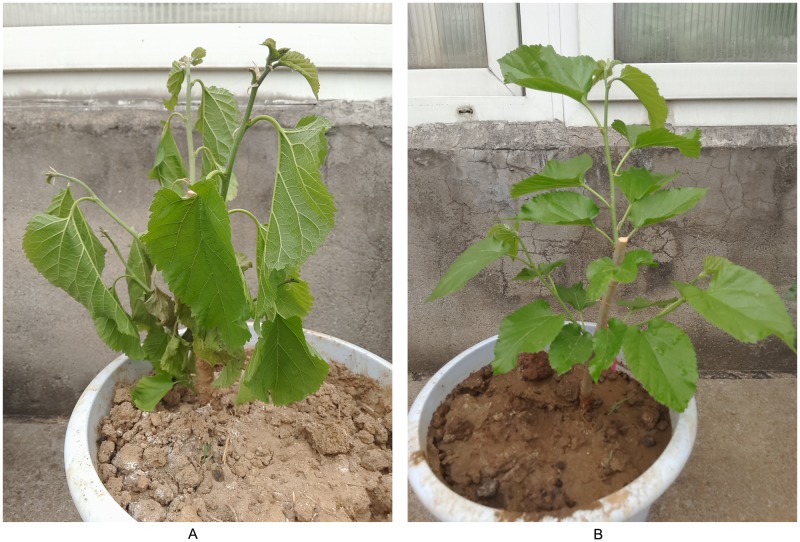
Effects of drought stress on phenotypic alterations in mulberry sapling. (A) After drought treatment for 15 days. (B) Control mulberry sapling.

The mulberry species (*Morus alba*), ‘Yu711’, was obtained from the National Mulberry Gene Bank in Zhenjiang, Jiangsu, China. The plants were grown in a greenhouse under a photo period of 14 h light/10 h dark at 25°C day/20°C night. Mulberry cuttings were grafted to rootstocks. The grafted nurseries were planted in 35 cm diameter pots containing loam soil with a nursery per pot. The grafted plants were randomly grouped when new shoots had grown to 20 cm in length. One group was used as the control, and the other one was treated with drought stress for 5 d, 10 d, and 15 d (Fig 1). Each grouping contained three replicates. Young leaves were collected from the same position of the plants and immediately frozen in liquid nitrogen and stored at -80°C for RNA extraction.

### Construction of the degradome library and sequencing

All centrifuge tubes and pipette-tips were RNase-free or were treated with DEPC. All buffers were RNase free or prepared using DEPC-treated ddH_2_O. Total RNA of each sample was extracted by Trizol (Life Technology, USA) according to the manufacturer’s instructions. For the same grouping of treatments, equal amounts of the RNA from each time point were mixed together. The RNA degradome library was constructed as previously described [5,37]. In brief, poly (A) RNA was isolated from approximately 200 μg of total RNA using the Oligotex mRNA mini kit (Qiagen, USA). A 5’-RNA adapter containing a *Mme* I recognition site in its 3’ terminus was ligated to the poly (A) RNA possessing a free 5’-monophosphate by T4 RNA ligase (Takara, China). The ligation products were purified using the Oligotex mRNA mini kit and reverse transcribed using the Oligo (dT)_18_ primer and Superscript II reverse transcriptase (Invitrogen, USA). The first-strand cDNA was amplified for five cycles using Ex Taq DNA Polymerase (Takara, China) and the PCR products were digested with *Mme* I. Next, the digested products were ligated to a 3’-double-stranded DNA adaptor using T4 DNA ligase (Takara, China), and amplified by PCR for 20 cycles. The final PCR products were gel purified and subjected to SE51 sequencing using Illumina HiSeq2000 (Illumina Inc., San Diego, USA).

### Bioinformatics analysis and identification of miRNA targets

Two mulberry degradome libraries were sequenced using Illumina HiSeq 2000. Low quality nucleotide reads and clip adapter sequences were removed from the raw data using the Fastx-Toolkit. High quality 20–21 nucleotide-long reads were subjected to the CleaveLand pipeline for small RNA targets identification as previously described [38]. Briefly, the raw tags were first normalized to ‘tags per 100 million’ (TP100M). After preprocessing, the non-coding RNAs were removed and clean tags were generated, saved, and classified by the alignment to the database and remove the ncRNAs (no coding RNAs). Clean tags were mapped to mulberry complete reference genome (http://morus.swu.edu.cn/morusdb/). The sequences of rRNAs, tRNAs, snoRNAs and snRNAs were retrieved from the Rfam database (http://www.sanger.ac.uk/Software/Rfam/). Degraded sequences in the clean reads were identified as polyN when over 70% of the sequence was a single base. Next, distinct tags that perfectly matched mulberry cDNA or mRNA sequences by SOAP2.20 based on Genbank of NCBI (http://www.ncbi.nlm.nih.gov/), the known miRNAs by PAREsnip from miRBase (miRBase21.0: http://www.mirbase.org/), or miRNAs identified in other studies [3,39] were used to analyze miRNA-mRNA pairs. Alignments were then scored according to a previously described scheme developed for plant miRNA/target pairings [40]. All alignments with scores up to seven and no mismatches at the cleavage site (between the 10th and 11th nucleotide) were considered as candidate targets. CL and DL data were analyzed separately. The identified targets were grouped into five categories based on the relative abundance of the degradome tags mapping at the miRNA target site through the height of the degradome peak at each occupied transcript position. Category 0: >1 raw tags at the position, abundance at the position was equal to the maximum on the transcript, and there was only one maximum on the transcript; category 1: >1 raw tags at the position, abundance at the position was equal to the maximum on the transcript, and there was more than one maximum position on the transcript; category 2: >1 raw tags at the position, abundance at the position was less than maximum but higher than the median for the transcript; category 3: >1 raw tags at the position, abundance at the position was equal to or less than the median for the transcript; category 4: only 1 raw tag at the position.

### Construction of miRNA-targets network

Identified targets and related miRNAs based on the analysis of degradome sequencing in DL and CL the profiles were used for building and visualizing miRNA-mRNA interaction global network using Cytoscape 3.2.1 software as previously described [41,42,43]. Subsequently, in order to further understand the regulatory relationships between drought responsed miRNAs and their target genes, we further filtered the target genes with P ≤ 0.05 in DL. Using these filtered interaction pairs, the specific network of miRNA-mRNA which *P* ≤ 0.05 and homologous to drought-related miRNA targets in DL were also constructed. In the network, nodes represent miRNAs or target genes. The network structure is formed of basic elements (target genes and miRNAs; named nodes) and the connections representing miRNA-target interactions (named edges). If two genes were annotated to be related, an edge was added between them in the network.

### Detection of the expression profiles of miRNAs and their targets by qRT-PCR

To determine the regulatory relationship between miRNAs and their targets, the expression profile of 12 drought responsive miRNAs and their targets were examined by stem-loop qRT-PCR as previously described [44,45]. Total RNA was isolated from the leaves of the contrast and treated plants at 5 d, 10 d, and 15 d under drought stress and with RNAiso plus (Takara, China) according to the manufacturer’s instructions. The first cDNA strand was synthesized from total RNA using the M-MLV-reverse transcriptase (RTase) (Takara, China) and miRNA specific stem-loop primers were designed according to the method described by Chen et al. [44]. Briefly, six nucleotides that paired with the 3’ end of the miRNA were linked to a stem-looped sequence (GTCGTATCCAGTGCAGGGTCCGAGGTATTCGCACTGGATACGAC) to synthesize the stem-loop reverse transcription primer. The reactions contained 1 μg of total RNA, and each reaction was performed with 1 μ l20 μM gene-specific stem-loop primers. The RNA and primers were mixed with DEPC-treated water up to 6 μl, then After, incubated at 70°C for 10 min and immediately chilled on ice for more than 2 min. Then, 2.5 μl 5 × RT-Buffer, 0.5 μl 10 mM dNTP, 0.5 μl RNase Inhibitor and 0.5 μl M-MLV- RTase were added together to a total volume of 10 μl. Synthesis was performed at 42°C for 60 min and inactivation of the enzyme was performed at 72°C for 15 min. The product was then diluted 3-fold and 1 μl cDNA was used as the template to perform the qRT-PCR with each miRNA specific forward primer and universal primer. For target genes, 1 μg RNA and 1 μl 50 μM Oligo(dT)_18_ primer were mixed with DEPC-treated water to 17 μl, then incubated at 70°C for 10 min and after, ice-cooled immediately for more than 2 min. Then, 5 μl 5× RT-Buffer, 1.25 μl 10 mM dNTP, 0.75 μl RNase Inhibitor and 1 μl M-MLV RTase were added. The next steps were identical to the reverse transcription of miRNA. The product was then diluted 4-fold and 1 μl cDNA was used as the template to perform the qRT-PCR with each target gene primer. The primers for miRNAs, targets and β-actin (selected as a reference gene for normalization) were mixed with the FastStart Universal SYBR Green Master Mix kit (Roche, USA) based on the handbook, and 20 μl of the reaction mix was added to each well. Reactions were performed in LightCycler^®^ 96 Real-Time PCR System (Roche, USA) with thermal cycling parameters at 95°C for 600 s followed by 45 cycles of 95°C for 10 s, 59°C for 10 s, and 70°C for 10 s. During amplification, melting curves were constructed. The sequences of stem-loop reverse transcriptase primers, miRNA-specific PCR primers and target-specific PCR primers were listed in S1 and S2 Tables, respectively. All reactions were assayed in three biological replicates with three technical replicates. The relative expression differences of miRNAs and their targets were calculated by 2^-ΔΔCt^ method. Standard errors and standard deviations were calculated from replicates and significance was measured through one-way ANOVA Duncan’s multiple range test at the level of 0.01 < *P* ≤ 0.05 and *P* ≤ 0.01.

### Validation of targets by RLM-5’ RACE

In order to validate the cleavage sites of miRNA to target genes, a modified RLM-5’ RACE was performed using the FirstChoice RLM-RACE Kit (Ambion, USA) without calf intestine alkaline phosphatase (CIAP) and tobacco acid pyrophosphatase (TAP) treatments for six target genes in the drought stressed degradome library as previously described [46,47]. Total RNA was extracted from drought stressed mulberry leaves with RNAiso plus (Takara, China) as described by the manufacturer. Then, approximately 2 μg RNA was ligated with 5’ RACE adaptors (5’-GCUGAUGGCGAUGAAUGAACACUGCGUUUGCUGGCUUUGAUGAAA-3’) using T4 RNA ligase. The ligated mRNAs were then reverse-transcribed using random decamers via M-MLV RTase following the manufacturer’s instructions. Two rounds of 5’ RACE reactions were performed and the initial PCR was carried out using the RT reaction product, the 5’ RACE outer primer (5’-GCTGATGGCGATGAATGAACACTG-3’), and the gene-specific outer primer, Nested PCR was carried out using 1/10 of the initial PCR reaction product, the 5’ RACE inner primer (5’-CGCGGATCCGAACACTGCGTTTGCTGGCTTTGATG-3’) and gene-specific inner primer. The final PCR products were gel purified, cloned into the pMD18-T Vector (Takara, China), and sequenced. Each target was confirmed by at least four clones. Gene-specific PCR primers were designed from between 150 and 600 nucleotides from the 5’ end of the predicted target site and were listed in S3 Table.

### Functional annotations of the miRNA targets

To investigate the putative biological functions of target genes and biological processes possibly regulated by miRNAs in mulberry, Gene Ontology (GO) annotations, Kyoto Encyclopedia of Genes and Genomes (KEGG) pathway analysis (http://www.kegg.jp/kegg/pathway.html), and the Non-Redundant (NR) Protein Database [48–51] were employed to annotate and classify target genes using the DAVID gene annotation tool. For enrichment analysis, a hypergeometric distribution based statistical test (level of significance at 0.05) was used to reject the chances of randomness in the miRNA’s associations to target genes with their corresponding ontology term. The GO categorization results were listed as three independent hierarchies for biological process, cellular component, and molecular function. GO classification of the drought responsive miRNAs targets and the metabolic pathways of these genes were obtained based on KEGG analysis.

## Results

### Mulberry degradome library construction, sequencing, and sequence analysis

**Fig 2:**
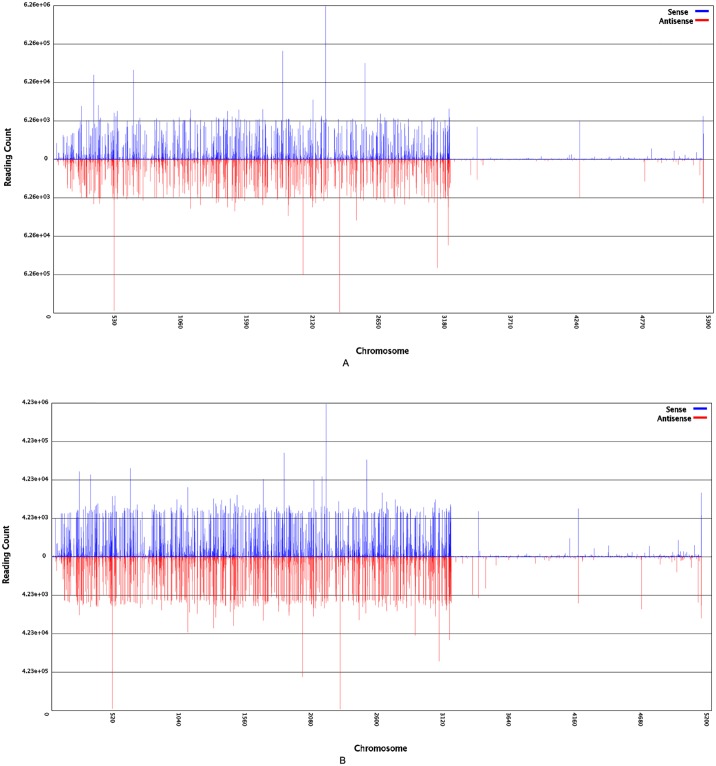
Chromosomes distribution of clean tags in CL and DL. The x axis indicates the chromosomes. The y axis indicates the number of tags that locate on each chromosome: area above 0 shown on blue is the number of tags on the sense strand of chromosome, whereas area below 0 shown in red is the number of tags on the antisense strand of chromosome. (A) CL. (B) DL.

In order to identify the miRNA targets in mulberry plants at a global level, two degradome libraries (drought library and contrast library) which captured the cleaved mRNAs were constructed for sequencing by Illumina sequencer. Our data have been deposited in the NCBI’s Gene Expression Omnibus (http://www.ncbi.nlm.nih.gov/geo/) with the accession number GSE84889. A total of 17,193,823 raw tags for CL and 20,200,629 tags for DL were obtained. After trimming low quality tags, adapter tags and N tags, there were 17,188,651 (99.97%) clean tags in CL and 20,192,466 (99.96%) clean tags in DL. Of those, there were 26,818,165 total common clean tags in CL and DL. There were 3,772,569 and 6,790,383 specific clean tags in CL and DL, respectively (Table 1). Of the 17,188,651 clean tags, 948,369 were identified as fragments of rRNAs, 348 were identified as fragments of tRNAs, 398 were identified as fragments of small nuclear RNA (snRNAs), and 1026 were identified as fragments of rRNAs, tRNAs, small nuclear RNA (snRNAs) and small nucleolar RNAs (snoRNAs) in CL, respectively using a BLASTN search against the Rfam database. Of the 20,192,466 clean tags, 693,817 were identified as fragments of rRNAs, 546 were identified as fragments of tRNAs, 612 were identified as fragments of small nuclear RNAs (snRNAs), and 1516 were identified as fragments of small nucleolar RNAs (snoRNAs) in DL, respectively (Table 1). The priority rule, Rfam > Genbank > Poly N, was used to map every unique degradome to the corresponding annotation, and map unannotated tags to reference genes (cDNA), classification of clean tags in CL and DL were listed in Table 1. The chromosomes distribution of clean tags was obtained by mapping clean tags to the complete mulberry reference genome using SOAP2.20, as shown in Fig 2. Finally, 10,132,806 (58.95%) and 9,353,151 (46.32%) clean tags in CL and DL were mapped to the genome, respectively. These data indicated that our two degradome libraries were of high quality and recovered most of the degraded mRNA targets that contained the sequence profile of miRNA-mediated cleavage and allowed us to conduct further analysis.

**Table 1:** Summary of mulberry degradome sequences by Illumina sequencing.

Category	CL		DL	
	Total reads	Unique reads	Total reads	Unique reads
***Statistics of degradome sequences by Illumina sequencing***				
**Raw tags**	17193823		20200629	
**Clean tags**	17188651(99.97%)		20192466(99.96%)	
**CL& DL clean tags**	26818165	954638	26818165	954638
**specific clean tags**	3772569	2572100	6790383	3042896
**Filter N tags**	129		167	
**Filter low quality tags**	5043		7996	
**Poly A**	44883(0.26%)	28835	83345(0.41%)	45317
***Mapping to genome***	10132806(58.95%)	1002456(28.42%)	9353151(46.32%)	955210(23.89%)
***Alignment to Rfam***				
**rRNA**	948369(5.52%)	9252(0.26%)	693817(3.44%)	10242(0.26%)
**tRNA**	348(0.00%)	134(0.00%)	546(0.00%)	151(0.00%)
**snRNA**	398(0.00%)	185(0.01%)	612(0.00%)	229(0.01%)
**snoRNA**	1026 (0.01%)	179 (0.01%)	1516 (0.01%)	206 (0.01%)
***Alignment to Genbank***				
**rRNA**	1369086 (7.97%)	6041 (0.17%)	737041 (3.65%)	5296 (0.13%)
**tRNA**	11 (0.00%)	3 (0.00%)	7(0.00%)	4 (0.00%)
***Classification of clean tags***				
**rRNA**	2255306 (13.12%)	14565 (0.41%)	1386260 (6.87%)	14847 (0.37%)
**tRNA**	349(0.00%)	135(0.00%)	547(0.00%)	152(0.00%)
**snRNA**	398(0.00%)	185(0.01%)	612(0.00%)	229(0.00%)
**snoRNA**	1026(0.01%)	179(0.01%)	151(0.00%)	206(0.00%)
**Poly N**	47969(0.28%)	31385(0.89%)	88680(0.44%)	49086(1.23%)
**cDNA sence**	2040655(11.87%)	923550(26.19%)	3250912(16.10%)	862437(21.57%)
**cDNA antisence**	801688(4.66%)	26629(0.76%)	1031032(5.11%)	33040(0.83%)
**other**	12041260(70.05%)	2530110 (71.74%)	14432907 (71.48%)	3037537 (75.99%)

Two degradome libraries were constructed, CL (contrast library) and DL (drought library). Total RNA were extracted from mulberry leaves grown in contrast or drought stress condition.

### Systematic identification of miRNA targets in mulberry

Confirmation of miRNA targets was a prerequisite to better understand the functional roles of miRNAs and may result in the discovery of new non-conserved miRNAs. In mulberry, conserved miRNA targets were previously investigated mainly by bioinformatics prediction and only a few miRNA targets have been experimentally validated [3,39]. High-throughput degradome sequencing was performed in this study to identify more miRNA targets in mulberry, particularly specific targets of drought-stress-responsive miRNAs. A characteristic feature of miRNA-guided cleaving was that the cleavage takes place precisely between the 10th and 11th nt from the 5’ of miRNA in the complementary region of the target transcript. Therefore, cleaved RNA targets should have distinct peaks of degradome tags at the predicted cleavage site relative to other regions of the transcript. Systematic identification of miRNA targets was accomplished by analyzing the 20 and 21 nt tags with the CleaveLand pipeline for miRNA target identification using the methods described above.

As listed in S4 and S5 Tables, 409 target genes of 30 conserved miRNA families and 990 target genes of 199 novel miRNAs were identified in CL. In DL, 373 target genes of 30 conserved miRNA families and 950 target genes of 195 novel miRNAs were identified. Based on the abundance of tags at the targets’ cleavage sites, the miRNA targets were categorized into five classes as described above. There were 10, 26, 522, 23 and 818 targets in categories 0, 1, 2, 3 and 4 respectively, in CL, and 26, 9, 708, 73 and 507 targets in categories 0, 1, 2, 3 and 4 respectively, in DL. Remarkably, a conserved miRNA family could target various numbers of genes ranging from 1 up to 54 and a target could be cleaved by 1–7 different conserved miRNA or miRNA family. Of those conserved miRNA families in DL, mno-miR156, mno-miR172 and mno-miR396 had the highest number of targets with 54, 52 and 41 unique transcripts, respectively (S5 Table), indicating that these three miRNA families may be in the center of the gene regulation networks. In contrast, fewer targets were identified for mno-miR2111, mno-miR4376, mno-miR4995, mno-miR827, mno-miR390 and mno-miR5523, indicating that these miRNAs might act in specialized pathways. The target gene XM_010104394.1, a squamosa promoter-binding-like protein, predicted to be involved in metabolic processes in response to stimulus and catalytic activity, was cleaved by six mno-miR156s and mno-miR535, indicating that this miRNA may play an important role in the process of growth, physiological metabolism and the corresponding stress of plants. Many of the target genes of the conserved miRNAs were classified as the transcription factors (TFs), such as NAC (a target of mno-miRn108-5p), ARF (a target of mno-miR160, mno-mi172, mno-miR156 et al.), NFY (a target of mno-miRn223-5p), MYB (a target of mno-miR159, mno-miR858, mno-miRn148-5p et al.), HD-ZIP (a target of mon-miR166), GRAS (a target of mno-miR171), and GRF (a target of mno-miR396), and so on. These TFs are known to regulate diverse aspects of plant growth, development, and biotic and abiotic stresses. Compared with *A*. *thaliana* and rice, many conserved miRNA targets were found to be conserved in mulberry, indicating that the miRNA-target relationship was evolutionarily conserved [27,30].

**Fig 3:**
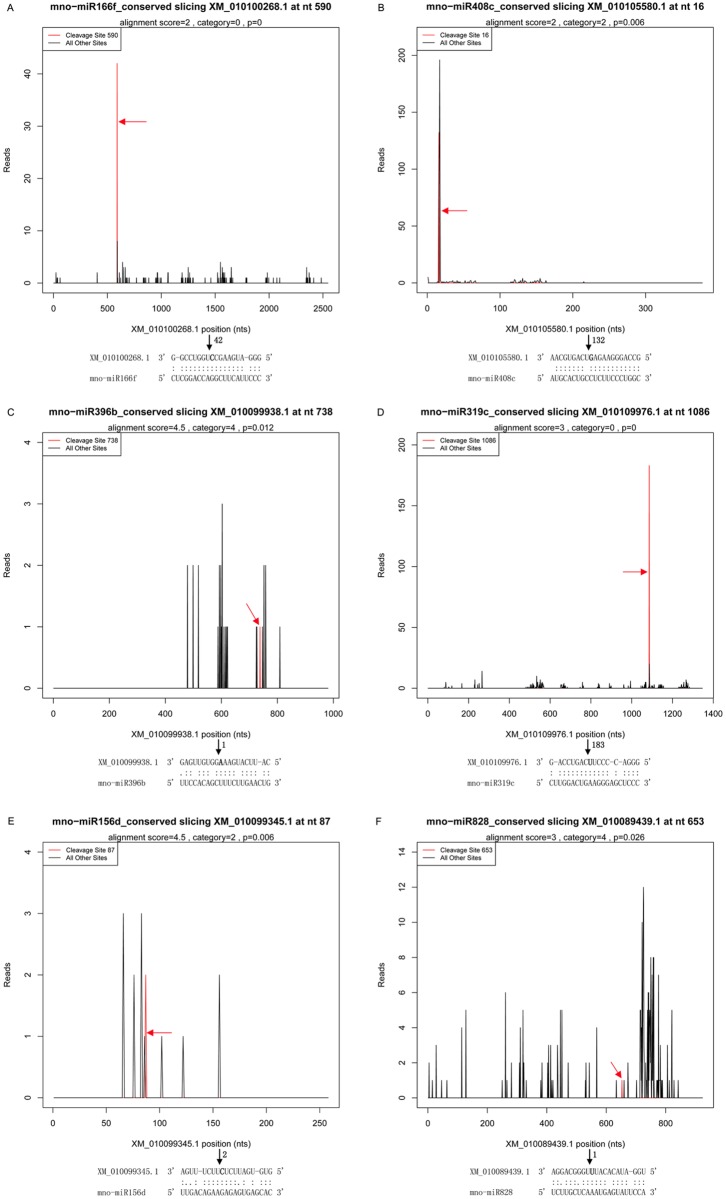
T-plots of the targets cleaved by miRNA in CL and DL. The T-plots show the distribution of 3’ end of the degradome tags within the full-lengthof the target mRNA sequence (bottom). The red line represents the cleaved target tags and is shown in red arrow. The alignment along with the detected cleavage frequencies (absolute numbers) are shown beside the black arrow and it shows the miRNA with a portion of its target sequence (top). The two dots indicate matched RNA base pairs; one dot shows a GU mismatch whereas none dot represent other types of mismatch. The bold letter (nucleotide) on the target transcript indicates the cleavage site showed by the blank arrow. (A) XM_010100268.1, a category 0 target for mno-miR166f in CL. (B) XM_010105580.1, a category 2 target for mno-miR408c in CL. (C) XM_010099938.1, a category 4 target for mno-miR396b in CL. (D) XM_010109976.1, a category 0 target for mno-miR319c in DL. (E) XM_010099345.1, a category 2 target for mno-miR156d in DL. (F) XM_010089439.1, a category 4 target for mno-miR828 in DL.

Of the 72 miRNA-mRNA pairs in CL and the 63 miRNA-mRNA pairs in DL with *P* ≤ 0.05, there were 29 and 27 target genes for 11 and 12 conserved miRNA families, respectively, and 36 and 27 target genes for 26 and 23 novel miRNAs, respectively (Tables 2 and 3). In CL, 72 miRNA-mRNA pairs were categorized into five classes as 6, 11, 14, 7 and 34 in category 0 to category 4, respectively. In miRNA-mRNA pairs of DL, 15 targets belonged to category 0, whereas the category 1–4 targets accounted for 6, 22, 8 and 12, respectively. The abundance of tags for each of them were presented in the form of target plots (T-plots) that plot the abundance of the tags relative to their position in the transcript [31]. For instance, six representative T-plots were shown, three from CL and another three from DL (Fig 3). In each of the six models, a clear peak for the absolute number of tags was found at the identified cleavage site for mno-miR166f, mno-miR408c, mno-miR396b, mno-miR319c, mno-miR156d and mno-miR828. Cleavage sites of all of the miRNAs were located in the coding DNA sequence (CDS) of the target genes identified by degradome sequencing in mulberry.

**Table 2:** Target genes of miRNAs in the mulberry CL library.

miRNA	Target	C.Site	Location	Score	Category	TP100M	P-Value
mno-miR156c	XM_010104394.1	1171	CDS	1	2	62	0
mno-miR160b	XM_010091672.1	1277	CDS	1	4	1	0
mno-miR162	XM_010112984.1	363	CDS	4	3	4	0.016
mno-miR164a	XM_010095292.1	652	CDS	3	4	1	0.026
mno-miR164a	XM_010099082.1	847	CDS	4.5	4	1	0.026
mno-miR166f	XM_010099828.1	560	CDS	2	0	202	0
mno-miR166f	XM_010100268.1	590	CDS	2	0	42	0
mno-miR166f	XM_010104104.1	587	CDS	2	0	5	0
mno-miR171a	XM_010090594.1	1283	CDS	0.5	2	2	0
mno-miR171c	XM_010112515.1	1256	CDS	1	2	2	0
mno-miR171d	XM_010112515.1	1253	CDS	1	2	2	0
mno-miR171e	XM_010112515.1	1253	CDS	2.5	2	2	0.002
mno-miR171h	XM_010112515.1	1253	CDS	2.5	2	2	0.004
mno-miR319c	XM_010109976.1	1086	CDS	3	0	101	0
mno-miR396b	XM_010088522.1	353	CDS	2	4	1	0.012
mno-miR396b	XM_010088832.1	342	CDS	4.5	4	1	0.012
mno-miR396b	XM_010091229.1	2641	CDS	4.5	4	1	0.012
mno-miR396b	XM_010091257.1	1117	CDS	4.5	4	1	0.012
mno-miR396b	XM_010092942.1	480	CDS	3.5	4	1	0.012
mno-miR396b	XM_010094796.1	248	CDS	4.5	4	1	0.012
mno-miR396b	XM_010097895.1	612	CDS	4	4	1	0.012
mno-miR396b	XM_010098038.1	90	CDS	4.5	4	1	0.012
mno-miR396b	XM_010099023.1	2606	CDS	4	4	1	0.012
mno-miR396b	XM_010099938.1	738	CDS	4.5	4	1	0.012
mno-miR396b	XM_010103804.1	840	CDS	3.5	4	1	0.012
mno-miR396b	XM_010104482.1	4656	CDS	4.5	4	1	0.012
mno-miR396b	XM_010104690.1	654	CDS	3.5	4	1	0.012
mno-miR396b	XM_010104691.1	777	CDS	3.5	4	1	0.012
mno-miR396b	XM_010106362.1	630	CDS	4	4	1	0.012
mno-miR396b	XM_010111944.1	992	CDS	4	4	1	0.012
mno-miR408c	XM_010105580.1	16	CDS	2	2	132	0.006
mno-miR4376	XM_010106326.1	22	CDS	3	4	1	0.012
mno-miR535	XM_010104394.1	1169	CDS	3	2	4	0.032
mno-miRn119-5p	XM_010114494.1	1031	CDS	4.5	1	3	0.03
mno-miRn120-3p	XM_010090594.1	1283	CDS	1	2	2	0
mno-miRn144-5p	XM_010102397.1	573	CDS	4.5	4	1	0.01
mno-miRn149-1-3p	XM_010096635.1	486	CDS	4.5	1	2	0.008
mno-miRn160-5p	XM_010094916.1	82	CDS	4.5	3	2	0.044
mno-miRn168-3p	XM_010091818.1	2177	CDS	4.5	1	3	0.048
mno-miRn171-1-3p	XM_010100675.1	920	CDS	4.5	3	2	0.032
mno-miRn175-5p	XM_010097012.1	36	CDS	4	0	3	0.01
mno-miRn178-1-3p	XM_010096254.1	531	CDS	4	3	2	0.04
mno-miRn178-1-3p	XM_010107694.1	147	CDS	4	3	2	0.04
mno-miRn200-3p	XM_010099010.1	2223	CDS	4	2	2	0.046
mno-miRn204-3p	XM_010089422.1	1789	CDS	2.5	4	1	0.006
mno-miRn204-3p	XM_010093987.1	3046	CDS	2.5	4	1	0.006
mno-miRn204-3p	XM_010106433.1	1396	CDS	4.5	4	1	0.006
mno-miRn205-5p	XM_010092962.1	254	CDS	3	4	1	0.048
mno-miRn205-5p	XM_010097789.1	586	CDS	4.5	4	1	0.048
mno-miRn205-5p	XM_010108021.1	551	CDS	3.5	4	1	0.048
mno-miRn207-3p	XM_010102455.1	1333	CDS	4.5	3	2	0.022
mno-miRn208-3p	XM_010090819.1	1125	CDS	4	0	3	0.018
mno-miRn219-3p	XM_010114286.1	358	CDS	4	1	2	0.032
mno-miRn32-1-3p	XM_010099111.1	2815	CDS	4.5	1	3	0.03
mno-miRn33-1-5p	XM_010099185.1	1347	CDS	4.5	1	2	0.032
mno-miRn33-1-5p	XM_010107920.1	1435	CDS	3.5	1	2	0.032
mno-miRn48-5p	XM_010102625.1	38	CDS	3.5	2	2	0.04
mno-miRn55-1-5p	XM_010102455.1	1333	CDS	4.5	3	2	0.032
mno-miRn67-3p	XM_010095642.1	1140	CDS	2	4	1	0.008
mno-miRn67-3p	XM_010099023.1	1649	CDS	4	4	1	0.008
mno-miRn67-3p	XM_010100444.1	34	CDS	4	4	1	0.008
mno-miRn67-3p	XM_010103502.1	405	CDS	4	4	1	0.008
mno-miRn67-3p	XM_010106088.1	2600	CDS	4	4	1	0.008
mno-miRn67-3p	XM_010108206.1	1549	CDS	4	4	1	0.008
mno-miRn69-3p	XM_010101296.1	870	CDS	4.5	1	3	0.024
mno-miRn70-1-5p	XM_010101296.1	870	CDS	3.5	1	4	0.002
mno-miRn74a-1-3p	XM_010090508.1	315	CDS	3.5	1	4	0.002
mno-miRn74b-3p	XM_010090508.1	315	CDS	4.5	1	4	0.028
mno-miRn81-3p	XM_010106888.1	853	CDS	3	4	1	0.02
mno-miRn99-3p	XM_010107049.1	24	CDS	3.5	2	5	0.046
mno-miRn99-3p	XM_010107437.1	74	CDS	4.5	2	10	0.046
mno-miRn99-3p	XM_010107438.1	74	CDS	4.5	2	10	0.046

C.site (Cleavage site): Nucleotide number from 5’ end of cDNA; CDS: Coding Sequence; Score: mapping score of mRNA-miRNAs pair; Category: the “category” of this cleave site; TP100M: Transcripts per 100 million; *P*-value ≤ 0.05 using Cleaveland pipeline.

**Table 3:** Target genes of miRNAs in mulberry DL library.

miRNA	Target	C.Site	Location	Score	Category	TP100M	P-Value
mno-miR156c	XM_010088675.1	943	CDS	1	2	4	0
mno-miR156d	XM_010090407.1	493	CDS	2	2	87	0.006
mno-miR156d	XM_010092101.1	158	CDS	4.5	2	3	0.006
mno-miR156d	XM_010092348.1	757	CDS	2	2	87	0.006
mno-miR156d	XM_010092557.1	198	CDS	4	2	5	0.006
mno-miR156d	XM_010099345.1	87	CDS	4.5	2	2	0.006
mno-miR156d	XM_010101121.1	278	CDS	4	2	4	0.006
mno-miR156d	XM_010104394.1	1171	CDS	1	2	52	0.006
mno-miR156d	XM_010114085.1	988	CDS	2	2	10	0.006
mno-miR156f	XM_010090407.1	492	CDS	1	2	7	0
mno-miR156f	XM_010092348.1	756	CDS	1	2	7	0
mno-miR156f	XM_010104394.1	1170	CDS	2	2	10	0.004
mno-miR156g	XM_010090407.1	492	CDS	0	2	7	0
mno-miR156g	XM_010092101.1	157	CDS	2.5	4	1	0.01
mno-miR156g	XM_010105976.1	1681	CDS	4.5	4	1	0.01
mno-miR156g	XM_010110753.1	129	CDS	4.5	4	1	0.01
mno-miR156g	XM_010111722.1	158	CDS	4	4	1	0.01
mno-miR160b	XM_010105965.1	1316	CDS	1	2	4	0
mno-miR166c	XM_010099828.1	561	CDS	1	2	2	0
mno-miR166f	XM_010099828.1	560	CDS	2	0	126	0
mno-miR166f	XM_010100268.1	590	CDS	2	0	25	0.002
mno-miR171a	XM_010090594.1	1283	CDS	0.5	2	6	0
mno-miR172c	XM_010093529.1	16	CDS	4	0	5	0.02
mno-miR172e	XM_010114499.1	593	CDS	4	0	5	0.018
mno-miR319c	XM_010093013.1	964	CDS	3	4	1	0.03
mno-miR319c	XM_010098565.1	37	CDS	4.5	4	1	0.03
mno-miR319c	XM_010109976.1	1086	CDS	3	0	183	0
mno-miR319c	XM_010114597.1	300	CDS	3	3	2	0.004
mno-miR396b	XM_010111150.1	1142	CDS	4	1	2	0.04
mno-miR399d	XM_010100088.1	326	CDS	2.5	3	3	0
mno-miR399e	XM_010100088.1	326	CDS	3.5	3	3	0.002
mno-miR399f	XM_010100088.1	326	CDS	3	3	3	0.004
mno-miR408c	XM_010095399.1	2180	CDS	4	1	6	0.01
mno-miR4376	XM_010106326.1	22	CDS	3	2	5	0.002
mno-miR535	XM_010104394.1	1169	CDS	3	2	3	0.048
mno-miR828	XM_010089439.1	653	CDS	3	4	1	0.026
mno-miRn11-3p	XM_010095698.1	1333	CDS	3	2	4	0.016
mno-miRn114-5p	XM_010112092.1	102	CDS	4	1	3	0.016
mno-miRn118-5p	XM_010101413.1	2530	CDS	3	2	2	0.006
mno-miRn120-3p	XM_010090594.1	1283	CDS	1	2	6	0
mno-miRn121-3-5p	XM_010105099.1	692	CDS	4.5	0	10	0.042
mno-miRn122-5p	XM_010093417.1	591	CDS	4	3	2	0.034
mno-miRn14-1-5p	XM_010097134.1	1342	CDS	4.5	0	7	0.028
mno-miRn144-5p	XM_010102397.1	573	CDS	4.5	2	2	0.006
mno-miRn150-3p	XM_010114658.1	421	CDS	3.5	3	3	0.002
mno-miRn157-5p	XM_010110553.1	2398	CDS	4	0	11	0.032
mno-miRn166-3p	XM_010102826.1	998	CDS	3.5	0	6	0.002
mno-miRn17-1-5p	XM_010097045.1	984	CDS	3	4	1	0.044
mno-miRn17-1-5p	XM_010100274.1	1122	CDS	4.5	4	1	0.044
mno-miRn17-1-5p	XM_010105164.1	337	CDS	4.5	4	1	0.044
mno-miRn179-5p	XM_010102516.1	328	CDS	4	3	2	0.042
mno-miRn193-5p	XM_010113910.1	612	CDS	3.5	0	5	0.004
mno-miRn202-1-3p	XM_010092821.1	863	CDS	4.5	1	4	0.034
mno-miRn202-1-3p	XM_010093270.1	1167	CDS	3.5	3	2	0.004
mno-miRn2-1-5p	XM_010095892.1	1059	CDS	4.5	0	24	0.03
mno-miRn220-5p	XM_010113440.1	797	CDS	4	4	1	0.034
mno-miRn238-5p	XM_010104930.1	493	CDS	4	0	15	0.046
mno-miRn25-1-3p	XM_010113161.1	77	CDS	4	0	5	0.03
mno-miRn260-5p	XM_010110513.1	1280	CDS	4.5	1	4	0.024
mno-miRn37-3p	XM_010114015.1	477	CDS	4	4	1	0.048
mno-miRn46-5p	XM_010093108.1	3876	CDS	4.5	0	19	0.014
mno-miRn62-1-5p	XM_010092824.1	18	CDS	4.5	1	4	0.026
mno-miRn79-1-3p	XM_010091565.1	275	CDS	4	0	7	0.016

The same as Table 2.

### Identification of miRNA targets in response to drought stress

**Fig 4:**
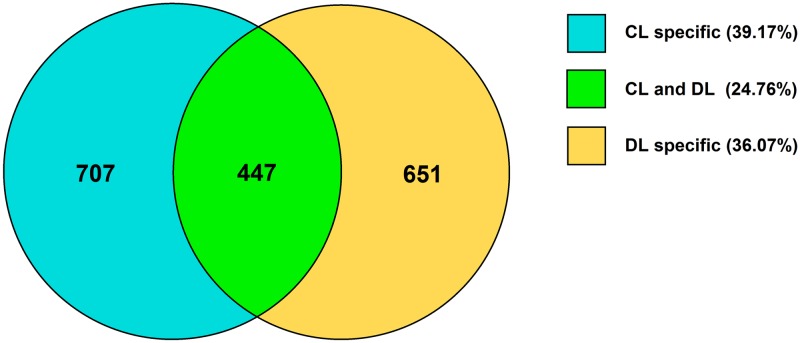
Venn chart of the target genes detected in the CL and DL libraries. The number and percentage was the quantity and proportion of target genes in CL specific or DL specific or both CL and DL, respectively.

All reference genes containing degradation sites were listed in S6 Table. In total, 1,154 and 1,098 targets were investigated in CL and DL, respectively. And 651 (36.07%) targets were found only in DL (Fig 4). Of the target genes with *P* ≤ 0.05 in DL, the mno-miR156 family conferred the highest number of targets with 17 (45.95%) of the conserved miRNA families (Table 3), and only one miR156-mRNA pair belonged to CL (Table 2), indicating that the miR156 family and their target genes played a key important function in responding to drought stress in mulberry. mno-miR156 targeted a number of mulberry genes annotated as SPB, vesicle transport protein GOT1A, ARF, GRAM domain-containing protein, and chloroplast stem-loop binding protein. Other targete genes of miRNAs were annotated as TFs TCP4, tubulin gamma-1 chain, pentatricopeptide repeat-containing protein, NEDD8-activating enzyme E1 catalytic subunit, auxilin-related protein 2, arginine/serine-rich-splicing factor RSP31, polyamine transporter At3g13620, AT-rich interactive domain-containing protein, mitochondrial import inner membrane translocase subunit, and ATP binding protein.

### GO, KEPP and NR analysis of miRNA target genes in mulberry

The identified targets for miRNAs in the two mulberry degradome libraries were annotated by searching NR databases. By blasting 1800 identified target genes against the NR database, 35,841 functional module structures with the domain sequences were obtained, as shown in S6 and S7 Tables. The highest percentage of the target genes (50.83%) had a global similarity of 60%-80% (Table 4), most of which were targeted by the conserved miRNAs. In the NR annotation by species distribution, the target genes in *M*. *multicaulis* had the highest percentage (45.94%) showing similar protein function with *Prunus persica*, followed by 13.35% in *Vitis vinifera*, suggesting that *M*. *multicaulis* had a similar molecular basis for drought stress with these two species (Table 5).

**Table 4:** Global analysis of the homology in the target genes by NR annotation.

similarity	gene numbers	percentage
20%-40%	37	2.06%
40%-60%	243	13.52%
60%-80%	914	50.83%
80%-95%	541	30.09%
95%-100%	63	3.50%

**Table 5:** Functional similarity in the targets with other species.

species	gene numbers	percentage
*Prunus persica*	826	45.94%
*Vitis vinifera*	240	13.35%
*Fragaria vesca subsp*	193	10.73%
*Ricinus communis*	167	9.29%
*Populus trichocarpa*	96	5.34%
*Cucumis sativus*	80	4.45%
*Glycine max*	74	4.12%
other	122	6.78%

To better understand the metabolic pathways of miRNA target genes in mulberry, KEGG was employed to predict the function of those targets, and 119 pathways were involved (S7 and S8 Tables). Importantly, most target genes were associated with metabolic pathways (318 genes), followed by biosynthesis of secondary metabolites (136 genes), plant hormone signal transduction (89 genes), RNA transport (78 genes), and plant-pathogen interaction (76 genes), as shown in S8 Table. The plant hormone signal transduction pathway includes many TFs and key enzymes, such as HD-ZIP and shaggy-related protein kinase (*SK*), which play important roles under stress response and abiotic stress.

**Fig 5:**
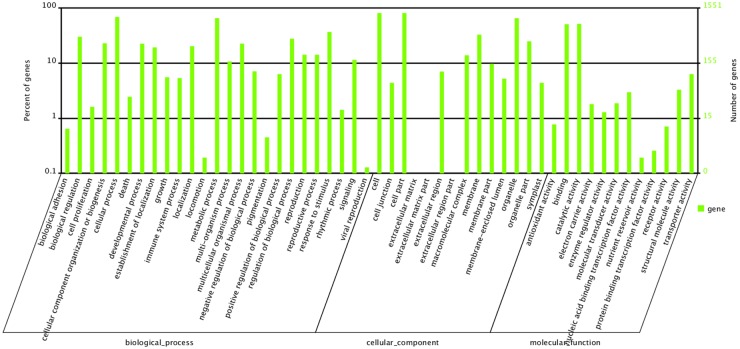
GO analysis of miRNA target genes identified in DL. The digitals on the left y-axis and right y-axis shows the percentage and enrichment of miRNA targets, respectively. Only the identified target genes for miRNAs by degradome sequencing were considered.

A total of 1,561 miRNA targets identified in DL were classified into three categories according to GO analysis: biological process, cellular component and molecular function, which were classified into 24, 14 and 12 terms respectively, as shown in Fig 5. More than 1000 targets were found to be involved in the cell, cell part, cellular process, metabolic process and organelle. The target genes were mostly associated with stimulus (including 568 genes), regulation of biological process and biological regulation. The results suggested that the corresponding miRNAs regulated the expression of these target genes during drought stress in mulberry by affecting various TFs to induce or shut off specific metabolic networks during response to adversity.

### Overview of miRNA-target networks

**Fig 6:**
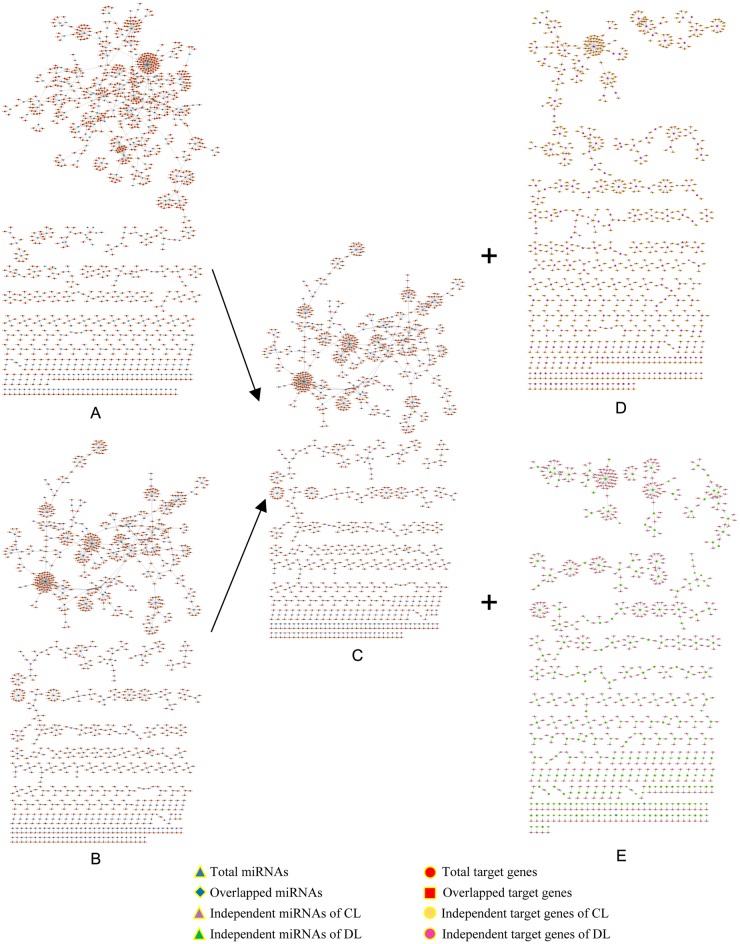
The global profiles of miRNA-target networks in CL and DL trials. The global profiles of CL and DL miRNA-target networks were constructed, respectively, and included the overlapped network and independent networks of CL and DL. The nodes represent miRNAs or target genes, and the edges represent the connection strength. (A) CL global network. (B) DL global network. (C) Overlapped network. (D) Independent network of CL. (E) Independent network of DL.

The consecutive miRNA-target networks of each library were constructed by Cytoscape 3.2.1 using the miRNAs and target genes. The network structure consists of the basic elements (genes and miRNAs; named nodes) and the connections representing miRNA-target interactions (named edges). To reveal the details of the network, the global networks were categorized into overlapped network and independent networks in CL and DL (Fig 6). Analysis of the nodes of the network, revealed that the independent networks consisted of 910 miRNA-mRNA pairs (65.05%) and 838 miRNA-mRNA pairs (63.34%) in CL and DL, respectively. This finding indicated that the interaction of miRNA-mRNA pairs differed between CL and DL, suggesting that the expression of numerous miRNAs may play roles in regulating the expression of target genes in response to drought stress.

**Fig 7:**
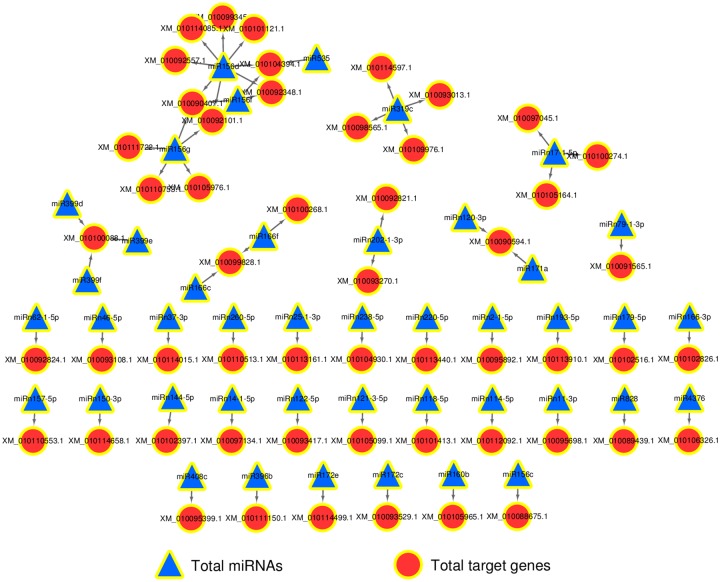
The specific profiles of miRNA-target networks in DL trials. The specific profiles of DL miRNA-target network were constructed using the target genes with *P* ≤ 0.05. The nodes represent miRNAs or target genes, and the edges represent the connection strength.

To increase our understanding of the regulatory role of drought-related miRNAs and their target genes, we constructed specific profiles of miRNA mediated interaction network using the target genes with *P* ≤ 0.05 in DL (Fig 7). This network contained 43 miRNAs and 53 genes, and revealed that many nodes were connected through miRNA-mRNA interaction data and formed a complex network. In this network, most of these targets identified by degradome sequencing were TFs, including ARF, SPB, HD-ZIP, MYB, TCP, SCL, GRAS and SPL. Targets analyzed by Nr annotation included diverse and important enzymes, such as NEDD8-activating enzyme E1 catalytic subunit, copperion-binding protein, type 2A protein phosphatase, transaldolase and CSP41A, suggesting that these genes may play important roles in the stress response.

### Detection of the expression of miRNA and their targets by qRT-PCR

To investigate the regulatory function of miRNAs on their target genes, the expression profiles of 13 miRNA-mRNA pairs (mno-miR166f for XM_010099828.1, mno-miR166f for XM_010100268.1, mno-miR166c for XM_010099828.1, mno-miR171a for XM_010090594.1, mno-miR319c for XM_010109976.1, mno-miR535 for XM_010104394.1, mno-miR4376 for XM_010106326.1, mno-miR156d for XM_010092101.1, mno-miR172a for XM_010106115.1, mno-miRn120-3p for XM_010090594.1, mno-miRn144-5p for XM_010102397.1, mno-miRn46-5p for XM_010093108.1, and mno-miRn202-1-3p for XM_010093270.1) were selected with a *P* value ≤ 0.05 as criteria from DL for stem-loop qRT-PCR. Among these, eight target genes were cleaved by eight conserved miRNAs and four target genes were cleaved by four novel miRNAs. With the exception of one miRNA-mRNA pair (mno-miR172a for XM_010106115.1) with *P* value > 0.05, the remaining 12 pairs had *P* values lower than 0.05.

**Fig 8:**
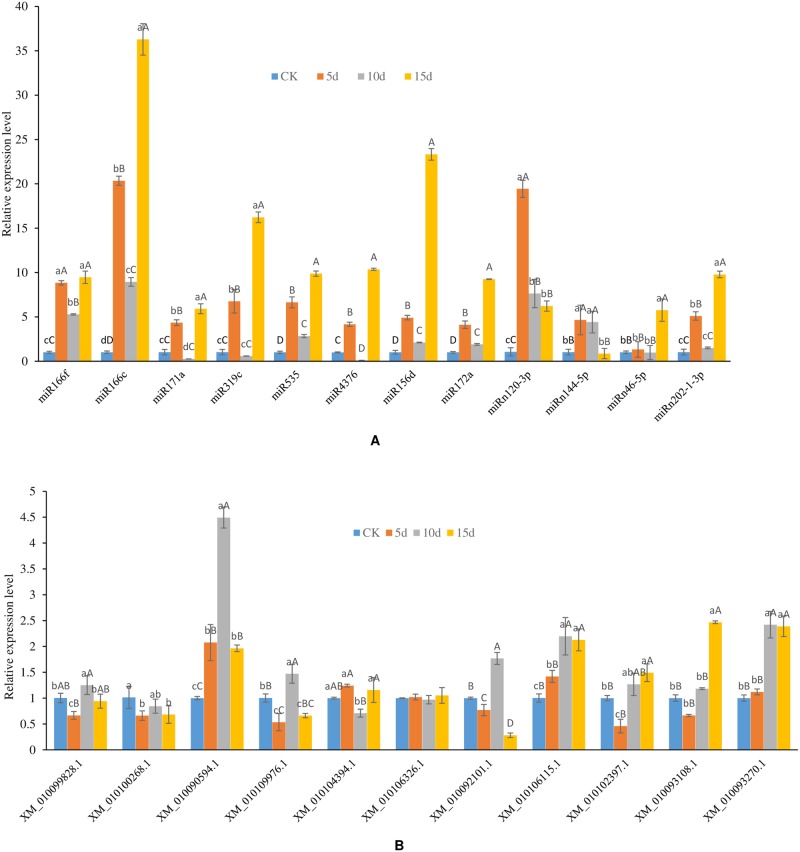
Expression analysis of 13 miRNA-mRNA pairs under drought stress with different processing time. The expression levels of miRNAs and their mRNA targets were normalized to the level of β-actin. The x axis represents different miRNA (A) or mRNA targets (B), and the y axis represents the relative expression level of miRNAs (A) or mRNA targets (B). The results are mean ± SD of the triplicates of three biological replicates. Letter superscripts above bar indicate the significant difference of the changes between different drought stress time with the level of *P* > 0.05 (shown as the same or no letter superscripts), 0.01 < *P* ≤ 0.05 (shown as different small letter superscripts) and *P* ≤ 0.01 (shown as different capital letter superscripts). (A) Expression profile of miRNAs. (B) Expression profile of mRNA targets.

As shown in Fig 8A, the expression levels of miR166f, miR166c, miR171a, miR319c, miR156d, miR172a and miRn202-1-3p decreased to the lowest value after 10 d under drought stress and were higher at both 5 d and 15 d. Within the same time period, their corresponding targets XM_010099828.1, XM_010100268.1, XM_010090594.1, XM_010109976.1, XM_010092101.1, XM_010106115.1, and XM_010093270.1 (Fig 8B), exhibited apparently the opposite expression profile. miRn144-5p and its target XM_010102397.1 also exhibited the opposite expression profile. miRn144-5p, however, exhibited down-regulation under drought treatment from 5 d to 15 d, and its target XM_010102397.1 exhibited up-regulation. The expression correlation of these nine miRNAs and their targets illustrated that these miRNAs negatively regulated their targets. miR535 and miR4376 exhibited the same expression profile as their targets, XM_010104394.1 and XM_010106326.1, which declined from 5 d and then increased on 15 d. Two miRNA-mRNA pairs, miRn120-3p for XM_010090594.1 and miRn46-5p for XM_010093108.1, showed non-significant regulation, suggesting that one miRNA may regulate multiple target genes or one gene may be regulated by multiple miRNAs. With increased drought stress time, the expression levels of miRNAs appeared to change significantly, and the expression profiles of many miRNAs increased first, then decreased and increased again. Further studies will be needed to shed light on the regulation network of these miRNAs and their targets in response to drought. Over expression or repressing expression of these miRNAs under drought stress may help to elucidate the regulatory mechanism.

### Determination of six miRNA targets by RLM-5’ RACE

To further confirm the degradome data, RLM-5’ RACE experiments were successfully employed to validate targets of conserved and novel miRNAs.

**Fig 9:**
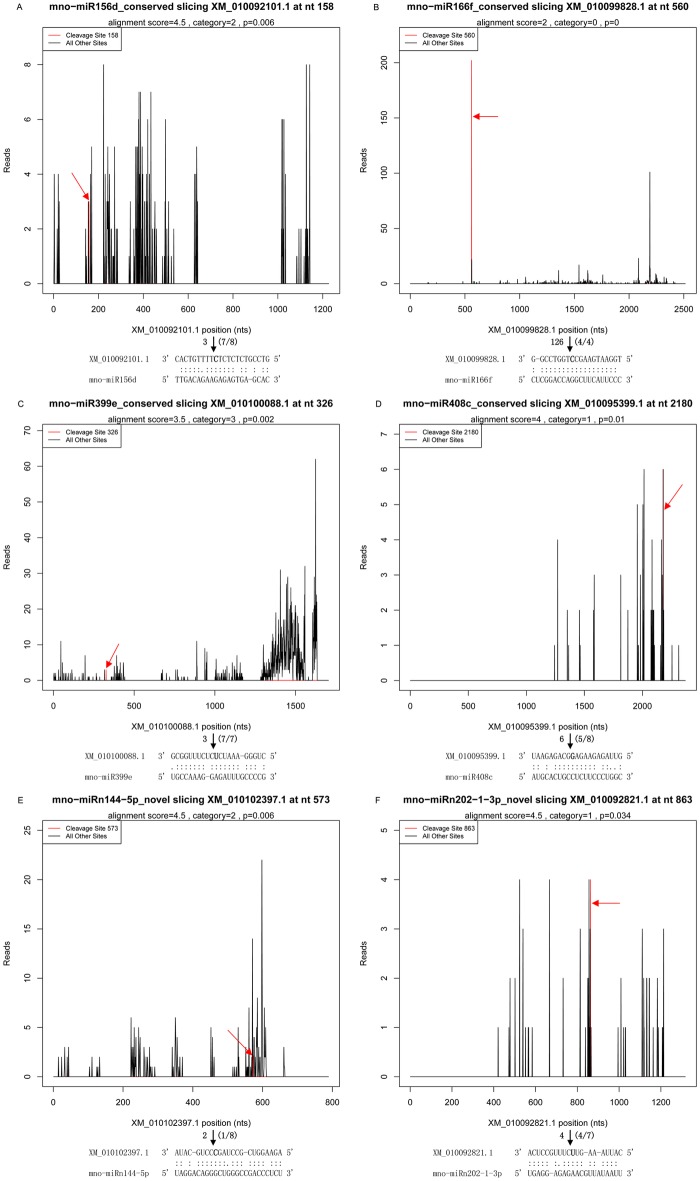
Validation of target genes cleaved by four conserved miRNAs and two novel miRNAs in DL. The nucleotide on the target transcript from 3’ end indicated the cleavage site detected in the degradome. The red line represents the cleaved target tags and is shown in red arrow in T-plots. The cleavage site is shown as a bold letter (nucleotide) and absolute numbers of signature sequences are shown above the nucleotide with the bold letter. The black arrow shows a site verified by gene-specific RLM 5’-RACE and the cleavage frequency as determined at the indicated position is shown in parentheses. The two dots indicate matched RNA base pairs; one dot shows a GU mismatch whereas none dot represent other types of mismatch. (A) XM_010092101.1, a category 2 target for mno-miR156d. (B) XM_010099828.1, a category 0 target for mno-miR166f. (C) XM_010100088.1, a category 3 target for mno-miR399e. (D) XM_010095399.1, a category 1 target for mno-miR408c. (E) XM_010102397.1, a category 2 target for mno-miRn144-5p. (F) XM_010092821.1, a category 1 target for mno-miRn202-1-3p.

Four target genes for conserved miRNAs in category 2, 0, 3, 1, which were XM_010092101.1 for mno-miR156d, XM_010099828.1 for mno-miR166f, XM_010100088.1 for mno-miR399e, and XM_010095399.1 for mno-miR408c, respectively, were successfully validated by RLM 5’-RACE experiments (Fig 9A–9D). Additionally, two target genes for novel miRNAs in category 2 and 1, which were XM_010102397.1 for mno-miRn144-5p and XM_010092821.1 for mno-miRn202-1-3p, respectively, were also amplified and were cleaved at the same position found in the degradome library (Fig 9E and 9F).

## Discussion

### High-throughput sequencing of RNA degradome in mulberry

Small RNAs play important and fundamental roles in response to adversity, especially in drought stress response [19,52]. A huge number of miRNAs and siRNAs have been identified by cloning and deep sequencing in higher plants [33,39,53,54]. It is the pivotal problem to find target genes for interpretation of miRNA function. *Arabidopsis*, rice, maize and soybean miRNA targets have been widely studied by high throughput sequencing [27,30,55,56]. In mulberry, miRNA targets were previously investigated mainly via bioinformatics prediction [3,39]. However, this method yields a high level of mismatches in miRNA:target pairing and is laborious, time-consuming and costly for verification of every single predicted gene. Therefore, only a few conserved miRNA targets have been experimentally validated in mulberry [3]. To overcome these shortages, degradome analysis was used in this study. In order to study the regulation of gene expression during drought exposure, we constructed and sequenced two distinct degradome libraries using mulberry leaves under drought stress conditions. Intriguingly, the clean reads from two libraries match perfectly with the mulberry chromosomes and 862, 439 annotated genomic loci were identified in this study (these data have been deposited in the NCBI’s GEO with accession number GSE84889.). Thus, only a small fraction of the sequences are small RNA targets, implying that the vast majority of them are other types of RNA turnover products.

Importantly, our degradome data verified 50–80% of previously validated targets and predicted 15–30% of potential targets [30,31]. Six targets were validated employing RLM 5’-RACE. These targets were found to be cleaved at the same position with different cleavage frequency. Therefore, degradome sequencing, combining the advantages of high throughput deep sequencing and an effective computational approach, served as an efficient strategy to globally identify small RNA targets in plants. Previous studies have indicated that most miRNA targets are cut in the CDS in plants, which is different from animals [28]. Consistent with this finding, cleavage sites of all of the miRNAs were located in the CDS of the target genes identified by degradome sequencing in mulberry. As described by Md Shamimuzzaman [46], other genes may be potentially regulated by miRNA-guided cleavage in the UTR that have not been detected in our alignment analyses. Our identification of many targets captured by the degradome analysis was consistent with previous reports [5,14,27]. However, target genes of several conserved miRNAs (miR394, miR447, miR482, and miR530) and certain novel miRNAs (24.05%) were not detected through this degradome sequencing in mulberry. This phenomenon may result from a low level of some conserved/known non-conserved target genes of miRNAs. Alternatively, some miRNAs might inhibit target gene expression through translational repression [57–59]. To obtain more information on miRNA targets, degradome libraries from different tissues, organs, and developmental stages should be constructed and integrated with the complete mulberry genome sequence.

### Drought-responsive miRNAs and their targets in mulberry

Plant miRNAs play a role in the response to abiotic stress, such as drought, NaCl, and low temperature [11,20,55]. The key to determining the function of a miRNA is identifying its target genes. By deep degradome sequencing plants under drought stress, a total of 225 miRNAs targeting 1323 transcripts were detected, including 373 target genes of 30 conserved miRNA families and 950 target genes of 195 novel miRNAs. Under normal growth conditions, 229 miRNAs targeting 1399 transcripts were identified, including 409 target genes of 30 conserved miRNA families and 990 target genes of 199 novel miRNAs.

Among the conserved miRNA families in DL, we found that mno-miR156, mno-miR172 and mno-miR396 had the highest number of targets, which is consistent with previous research. Most of the mRNA target genes identified by degradome sequenceing were transcription factors, including ARF, SPB, HD-ZIP, MYB, TCP, SCL, GRAS, SPL and so on. These TFs played important roles in the response to abiotic stress in plants [30,34]. We found that miR408c targeted oxysterol-binding (OSBP)-related proteins (ORPs) and dehydration response element B (DREB), which are transcription factors involved in drought tolerance. Hajyzadeh et al. [60] reported that upon drought stress treatment, chickpea plants overexpressing miR408 showed normal growth, while other samples struggled with severe symptoms of stress. Meanwhile, the miR408-overexpressed plants exhibited a shorter plant height compared to vector control plants. Through Nr annotation, we also found that many target genes were important enzymes, such as *SK*, enzyme E1 catalytic subunit, the type 2 a protein phosphatase, transaldolase and CSP41A. These enzymes also play an important function during stress responses in plants [29]. Our results revealed that miR172a negatively regulates XM_010106115.1, a transcript expressed to *SK*, a plant hormone signal transduction pathway. Interestingly, different target genes were found in different tissues upon drought stress by Eldem et al. [24]. Of the miRNAs differentially expressed in response to drought stress, 60 miRNAs were found to be tissue-specific. 23 miRNAs were only expressed in leaf and 26 miRNAs were only expressed in roots under drought stress. Eldem et al. also found that in leaves, miR156 expression was 3-fold higher, but they did not detect a significant change in roots compared to control samples. The miR5281 was only expressed in leaf with 4-fold upregulation; in contrast, miR415 was downregulated 14-fold in the root but not expressed in the leaf. Target transcripts (137 for leaf control, 133 for leaf stress, 148 for root control and 153 for root stress) generated significant GO terms related to DNA binding and catalytic activities. In the present study, a number of novel miRNAs were found to target genes that: they were annotated as tubulin gamma-1 chain, pentatricopeptide repeat-containing protein, NEDD8-activating enzyme E1 catalytic subunit, auxilin-related protein 2, arginine/serine-rich-splicing factor RSP31, polyamine transporter At3g13620, AT-rich interactive domain-containing protein, mitochondrial import inner membrane translocase subunit, and ATP binding protein.

According to the expression levels of 12 miRNAs and 11 of their targets by qRT-PCR, we found that miR166f, miR166c, miR171a, miR319c, miR156d, miR172a, miRn202-1-3p and miRn144-5p negatively regulated their targets. miR535 and miR4376, however, showed the same trends in expression as their targets. miR156 and miR172 have been shown to regulate their target genes (SBPs and AP2) predominantly by inhibiting the translation of target genes in many plants [61,62]. miR171 has also been found to negatively regulate its target genes in *A*. *thaliana* [63]. We found that with increases in the time of drought stress, the expression levels of miRNAs appeared to change significantly, and the expression of many miRNAs increased first, then decreased and riseincreased again.

### Enrichment of transcription factors in miRNA targets

In plants, miRNAs target the genes involved in development and stress response, particularly TFs, metabolic transporters, and signal transduction factors. These genes can unravel a new dimension of the miRNA regulatory network [64]. Our analysis revealed that the majority of these target genes, classified into 51 different annotation categories (Fig 5), were conserved among species [46,65,66]. The target genes are obviously enriched in TFs and transcription regulatory activity, such as NAC, MYB, ARF, SPL, SCL, HD-ZIP, APS, NF-Y subunits, MADS-box, GRAS and TCP (S6 and S7 Tables). This phenomenon was similar to that found in *Arabidopsis*, rice, soybean, and maize [29,30,34]. A number of the TFs are known to regulate diverse aspects of plant growth, development and responses to environmental changes. For example, MYB family members, such as *MYB33* and *MYB101*, which are targeted by miR159 in rice and soybean, appear to play an important role in the response to abscisic acid (ABA) during seedling stress responses, suggesting that ABA-induced accumulation of miR159 is a homeostatic mechanism to direct *MYB33* and *MYB101* transcript degradation to desensitize hormone signaling during seedling stress responses. [46,67]. In this study, we detected a number of MYB family TFs regulated by mno-miR159, mno-miR858 and mno-miRn148-5p. miR159 expression is regulated by at least two different hormones, GA and ABA, and regulates the abundance of MYB mRNAs in response to drought stress [67]. Another important family of TFs, the HD-ZIP TF family, determines adaxial/upper cell fate [68]. Our findings indicate that the expression of miR166f increased significantly and its target gene XM_010100268.1, a HD-ZIP TF, was down-regulated during drought stress. According to GO analysis, XM_010100268.1 participates in biological regulation, metabolic process and nucleic acid binding transcription factor activity. NAC domain transcriptional regulator gene was the target of miR394 and miR482 [60], implying that NAC domain transcriptional regulators might also be regulated by other miRNAs. SPL TFs, which are unique to plants, are involved in embryonic development, plastochron length, leaf development, developmental phase transitions, flower and fruit development, fertility, apical dominance, anthocyanin biosynthesis, gibberellin response, light signaling and copper homeostasis. The miR156 family was found to target SPL in this study, consistent with a study involving cotton [60]. Auxin, an important phytohormone, acts as a key player in plant development in higher plants [69]. As transducers of auxin signaling, ARF and TIR play vital roles in plant development under drought stress conditions, including shoot, root and flower formation [70–73]. We also identified miR160, miR172 and some novel miRNAs involved in auxin signaling. NF-Y subunits are known to control a variety of important agronomical traits, including drought tolerance, flowering time, and seed development [74]. In *Arabidopsis*, overexpression of a miR169-resistant NF-YA5 transgene significantly improves drought resistance through the ABA-dependent pathway by promoting stomatal closure under drought stress [75]. In this study, we discovered two novel miRNAs, miRn71-3p and miRn223-5p, that targeted NF-Y. Four MADS-box genes have previously been validated as targets for miR444 variants in rice and they are regulated by several miR444 variants [29]. In mulberry, we found that miR169 targets the MADS-box.

### Regulation networks of miRNAs and targets during drought stress in mulberry

The rapid discovery of miRNAs and experimental evidence for miRNA interactions has ushered in a new era of miRNA research that focuses on networks other than individual miRNA, interactions. The conservation of resistance to adverse environments in plants can be assessed by the summation of gene expression and regulation network connectivity, which can also provide a new avenue for understanding molecular mechanisms and distinguishing functional processes in resistance progression. Here, the consecutive miRNA target networks of each library were constructed. The network structure consists of the basic elements (genes and miRNAs, named nodes) and the connections representing miRNA-target interactions (named edges). After construction of the network, we found that the topological profiles are more similar to a ‘Medusa’model [76], which consists of a regulatory core of hub nodes. This finding indicates that the kernel nodes of the network are determinants in the gene expression levels [40,77].

Generally, miRNA inhibits translation or induces mRNA degradation by binding to the CDS of target mRNAs in plants. As shown in the results, from the overlapped network and the independent network of miRNA-targets in CL and DL groups based on the degradome data (Figs 6 and 7), we identified differential miRNAs and their targets that are involved in drought stress. In degradom, 838 miRNA-mRNA pairs (63.34%), consisting of 272 miRNAs and 732 genes were found only in DL. The cluster results of the network showed that a miRNA can be applied to different mRNAs and a mRNA can also be regulated by a number of different miRNAs. Conversely, the changes in gene expression induced by a miRNA can also act to regulate this that miRNA or other miRNAs. This means that the interactions of miRNAs and mRNAs are not one-to-one, but can form interaction networks with cross regulation. Because of the cross regulation of target genes by miRNAs, small changes in the expression levels of miRNAs or mutations in the pairing sequences will produce serious consequences. This is similar to the cascade amplification effect in the signal transduction process [78]. Butz et al. found that the effect of miRNA on RCC has ‘divergent’ properties whereby the same miRNA targets multiple genes. It is also ‘convergent’ in nature, whereby multiple miRNAs have augmented effects on the same target genes [43]. Hua et al. [79] found that co-transfection of miR-20a and miR-361 had cumulative effects on inhibition of VEGF expression. Wu et al. [80] found that 28 miRNAs can significantly inhibit the expression of p21Cip1/Waf1. Xu et al. [78] constructed the miRNA functional networks (miRFNs) of soybean and found that miRFNs of soybean exhibit a scale-free, small world and modular architecture, with their degrees fit best to power-law and exponential distribution.

Among the miRNA-mRNA interaction pairs, the most important drought responsive target genes were TFs as described above. According to data analysis of miRNA target genes predicted by PicTar, Cui et al. [81] and Boyer et al. [82] also found that miRNAs are more inclined to regulate genes regulated by TFs. Here, we obtained 43 kernel miRNAs and 53 target genes from the network, which are highly correlated with the drought resistance in mulberry. Of the miRNA-mRNA pairs showing significant relationships (*P* ≤ 0.05), the mno-miR156 family conferred the highest number of targets of the 17 (45.95%) genes (Table 3), and one miR156-mRNA pair was found in CL (Table 2). In rice, the miR164-targeted NAC genes are known to be negative regulators of drought tolerance [83]. Our study revealed that miR156 targeted two target genes (XM_010104767.1 and XM_010092557.1), which belong to the NAC TFs family. Our study also demonstrated that miR156 can target SPL TF genes (miR156 for XM_010090407.1 and XM_010092348.1) and can regulate tolerance to recurring environmental stress as reported by Wang et al. [66] and Stief et al. [84]. These findings indicate that the miR156 family and their target genes may also play important roles in the response to drought stress in mulberry.

## Conclusions

This is the first comprehensive identification of drought responsive miRNAs and their targets in mulberry by degradome sequencing. This study provides a framework for further understanding the molecular mechanisms of resistance to drought in mulberry. Further experiments on the identified target genes are necessary to explore potential mechanisms of abiotic stress regulation in mulberry.

## Supporting information

S1 Table The stem-loop RT and qRT-PCR primer sequences for miRNAs expression analysis. (DOCX)

S2 Table The primer sequences for qRT-PCR analysis of target genes. (DOCX)

S3 Table The gene specific primer sequences for RLM-5’RACE of target genes. (DOCX)

S4 Table The information of miRNA-mRNA density in CL. (XLSX)

S5 Table The information of miRNA-mRNA density in DL. (XLSX)

S6 Table Nr annotation of ref genes in mulberry. (XLSX)

S7 Table Target genes blast by KEGG in mulberry. (XLSX)

S8 Table KEGG pathway participated by target genes in mulberry. (XLSX)
