# Supplementary material for: High throughput deep degradome sequencing reveals microRNAs and their targets in response to drought stress in mulberry (Morus alba)
Source: PLoS One. 2017 Feb 24;12(2):e0172883. doi: 10.1371/journal.pone.0172883 (PMC5325578; doi:10.1371/journal.pone.0172883)
Supplement: S1 Table — (DOCX) [file pone.0172883.s001.docx]

**Table S1 The stem-loop reverse transcription and** **qRT-PCR primer sequences for miRNAs expression analysis**

| gene | primer | sequence (5’-3’) |
| --- | --- | --- |
| β- actin | forward primer | TGGCTTATGTTGCCTTGGAC |
|  | reverse primer | GTTGGAAGAGGACTTGTGGG |
| universal | reverse primer | CAGTGCAGGGTCCGAGGTAT |
| mno-miR166f | stem-loop RT primer | GTCGTATCCAGTGCAGGGTCCGAGGTATTCGCACTGGATACGACGGGAAT |
|  | forward primer | CGTAGTACTCGGACCAGGCTT |
| mno-miR171a | stem-loop RT primer | GTCGTATCCAGTGCAGGGTCCGAGGTATTCGCACTGGATACGACGATATT |
|  | forward primer | TACGATTGATTGAGCCGTGC |
| mno-miR319c | stem-loop RT primer | GTCGTATCCAGTGCAGGGTCCGAGGTATTCGCACTGGATACGACAGGGAG |
|  | forward primer | GCTGTGATTGGACTGAAGGGA |
| mno-miR535 | stem-loop RT primer | GTCGTATCCAGTGCAGGGTCCGAGGTATTCGCACTGGATACGACGCGTGC |
|  | forward primer | GCGAGATGACAACGAGAGAG |
| mno-miR4376 | stem-loop RT primer | GTCGTATCCAGTGCAGGGTCCGAGGTATTCGCACTGGATACGACACAGCG |
|  | forward primer | CGAGGATACGCAGGAGAGAT |
| mno-miRn120-3p | stem-loop RT primer | GTCGTATCCAGTGCAGGGTCCGAGGTATTCGCACTGGATACGACGATATT |
|  | forward primer | AAGTACAGATTGAGCCGCGCC |
| mno-miRn144-5p | stem-loop RT primer | GTCGTATCCAGTGCAGGGTCCGAGGTATTCGCACTGGATACGACAGAGGG |
|  | forward primer | GCAATATAGGACAGGGCTGG |
| mno-miR156d | stem-loop RT primer | GTCGTATCCAGTGCAGGGTCCGAGGTATTCGCACTGGATACGACGTGCTC |
|  | forward primer | GCACGGTTGACAGAAGAGAG |
| mno-miR166c | stem-loop RT primer | GTCGTATCCAGTGCAGGGTCCGAGGTATTCGCACTGGATACGACGGAATG |
|  | forward primer | AACGTATCTCGGACCAGGCT |
| mno-miRn46-5p | stem-loop RT primer | GTCGTATCCAGTGCAGGGTCCGAGGTATTCGCACTGGATACGACAATAAA |
|  | forward primer | AGGCGGTCGAGTTGAGATGT |
| mno-miRn202-1-3p | stem-loop RT primer | GTCGTATCCAGTGCAGGGTCCGAGGTATTCGCACTGGATACGACAATTAT |
|  | forward primer | CGGCATTGAGGAGAGAACGT |
