# Supplementary material for: High throughput deep degradome sequencing reveals microRNAs and their targets in response to drought stress in mulberry (Morus alba)
Source: PLoS One. 2017 Feb 24;12(2):e0172883. doi: 10.1371/journal.pone.0172883 (PMC5325578; doi:10.1371/journal.pone.0172883)
Supplement: S2 Table — (DOCX) [file pone.0172883.s002.docx]

**Table S2 The primer sequences for** **qRT-PCR analysis of target genes**

| miRNA | Target gene | primer | sequence (5’-3’) |
| --- | --- | --- | --- |
|  | β- actin | forward primer  reverse primer | AAGTCATCACAATCGGAG  GGGAACATAGTTGAA CCA3 |
| mno-miR166f  mno-miR166c | XM_010099828.1 | forward primer  reverse primer | TCGTCGTCAACAGCTCATTC  CTCTGCTTCTCTCTGCATCTTC |
| mno-miR166f | XM_010100268.1 | forward primer  reverse primer | CAAGTTGAGGCTTTGGAGAGA  AGGCTCAATGTTGGAGAGAATAG |
| mno-miR171a  novel-miRn120-3p | XM_010090594.1 | forward primer  reverse primer | CCACCACCACCACCATAAA  TCAGAGCCCACATAGCAATTC |
| mno-miR319c | XM_010109976.1 | forward primer  reverse primer | CACCACCACAACAACAATCAG  ACGAAGAAGACGACGAAGTG |
| mno-miR4376 | XM_010106326.1 | forward primer  reverse primer | GGAGAAAGGCATTGGGAAGA  CTCTACCATCACCAAGCGTAAA |
| mno-miR535 | XM_010104394.1 | forward primer  reverse primer | CCGGTAGACTAACGCCAAATAA  GAAGAGAGAGAGCACGATGAAG |
| novel-miRn144-5p | XM_010102397.1 | forward primer  reverse primer | GGAGCGTCTCTTCTCCAAATAC  CGTATAGCATCTTCAGCATCCC |
| mno-miR156d | XM_010092101.1 | forward primer  reverse primer | CCATTTGTCCGTCTCTCTCTTT  ATGAGGACTCGTTTCTTGTCTG |
| novel-miRn202-1-3p | XM_010093270.1 | forward primer  reverse primer | GCAGTGTGACACCGGATTAT  GCGCTTAGTAGAGCTTGTGTAG |
| novel-miRn46-5p | XM_010093108.1 | forward primer  reverse primer | GAGGGCAAAGAGTTTCCATTTC  GAGTGTGTTCCGTCTTGGATAA |
| mno-miR172a | XM_010106115.1 | forward primer  reverse primer | CCAGAGGATGCCACTCATTTA  TGTGGCTTCACATCCCTATG |
