# Supplementary material for: High throughput deep degradome sequencing reveals microRNAs and their targets in response to drought stress in mulberry (Morus alba)
Source: PLoS One. 2017 Feb 24;12(2):e0172883. doi: 10.1371/journal.pone.0172883 (PMC5325578; doi:10.1371/journal.pone.0172883)
Supplement: S3 Table — (DOCX) [file pone.0172883.s003.docx]

**Table S3 The specific primer sequences for RLM-5’RACE of target genes**

| miRNA | Target gene | primer | sequence (5’-3’) |
| --- | --- | --- | --- |
|  |  | 5’RACE outer primer  5’RACE inner primer | GCTGATGGCGATGAATGAACACTG  CGCGGATCCGAACACTGCGTTTGCTGGCTTTGATG |
| mno-miRn202-1-3p | XM_010092821.1 | specific outer primer  specific inner primer | CTCCCTTGTCCTAGCCTGT  GCACAATCCAATACACAACCC |
| mno-miRn144-5p | XM_010102397.1 | specific outer primer  specific inner primer | CGGCGTTGATGTTGTGAATG  CGGTATCTGCCATATTCAGGAC |
| mno-miR399e | XM_010100088.1 | specific outer primer  specific inner primer | ACAGCATATTTTCGAGGACCA  ATTCATAAACCCATGCCGAGA |
| mno-miR166f | XM_010099828.1 | specific outer primer  specific inner primer | TGACGTAAAGCCGCCAT  CAGTACTTCAGGCACACTCC |
| mno-miR156d | XM_010092101.1 | specific outer primer  specific inner primer | CTTATAGATTCCGGCGCTGCT  CAGTCTTCCCTCCGGCACTCA |
| mno-miR408c | XM_010095399.1 | specific outer primer  specific inner primer | AATTCACCAAAGATATCGGGACA  CCATCCCATTTCCCCTGCTC |
